# Effect of extraction temperature and solvent type on the bioactive potential of *Ocimum gratissimum* L. extracts

**DOI:** 10.1038/s41598-020-78847-5

**Published:** 2020-12-10

**Authors:** Confidence Onyebuchi, Doğa Kavaz

**Affiliations:** 1 Bioengineering Department, Faculty of Engineering, Cyprus International University, Northern Cyprus via Mersin 10, 98258 Nicosia, Turkey; 2 Biotechnology Research Centre, Cyprus International University, Northern Cyprus via Mersin 10, 99258 Nicosia, Turkey

**Keywords:** Biotechnology, Cancer, Cell biology, Microbiology

## Abstract

*Ocimum gratissimum* is a shrub that belongs to the Lamiaceae family of plants*.* Despite the known biological activities and ethnomedicinal applications, comparative evaluation of the effects of different extraction techniques on the chemical and bioactive properties of *O. gratissimum* extracts has not yet been performed. This study adopted different analytical techniques to determine the effect of extraction temperature and solvent type on the phytochemical and bioactive properties of *O. gratissimum* extracts. Chemical profiling showed increased concentrations of compounds for both the ethanolic and methanolic extracts compared to the water extracts. The results also revealed that the extraction temperature had an effect on the total phenolic content and radical-scavenging properties of the different extracts. The antioxidant kinetic modeling achieved the best fit when using the second-order kinetic model. Methanolic extracts had the highest levels of antibacterial activity against *Escherichia coli*, *Bacillus cereus*, *Staphylococcus aureus*, and *Salmonella typhimurium*. At high concentrations, all extracts lowered the viability of the breast cancer cell line MDA-MB-231. In conclusion, the chemical and bioactive properties of all extracts showed significant dependence on the extraction temperature and solvent type. With proper extraction methods, they boast a wide range of promising applications in the medical, pharmaceutical, and food industries.

## Introduction

Microbial pollution is a global problem that is primarily caused by pathogenic bacteria such as *Bacillus cereus*, *Escherichia coli*, *Salmonella typhimurium*, and *Staphylococcus aureus*^1^. Recent studies have focused on the extraction of polyphenols from plants due to the antimicrobial^2^ and antioxidant^3^ effects of these compounds, as well as their ability to decrease food spoilage resulting from lipid oxidation without affecting the quality, safety, and freshness of the food product^4,5^. Moreover, a study by Shahidi and Zhong^6^ demonstrated the link between oxidative stress and the pathophysiology of several health conditions. *Ocimum gratissimum* L. often called clove basil or scent leaf, is indigenous to coastal, savannah, and tropical areas^7^, and is mostly found in tropical areas such as West Africa, Asia, Brazil, and India^8^. Ethnomedicinal applications have been reported for the plant due to its flowers and leaves being rich in polyphenols and other bioactive compounds^9^. Polyphenol isolation from plant sources can be achieved via several techniques. However, every technique has advantages and disadvantages. Hence, there is a need to ascertain which materials, techniques, and extraction conditions are optimal for polyphenol extraction, as the efficiency of the extraction process can affect the polyphenolic content and antioxidant capacity of the extract^10^. A universally acceptable protocol for extraction of polyphenols from plants would be difficult to establish due to the structural and compositional diversity of different plant phenolic compounds. Several variables, such as temperature and the nature of the solvent, may act independently or dependently to affect the extraction efficiency as well as the antioxidant capacity. Hence, there is a need to understand the mass transfer phenomenon for the extraction process using mathematical models. The use of kinetics and mathematical models facilitates the understanding of the mass transfer mechanism of the extraction process, which is essential for simulations, optimization, control, and design of the process and minimizes the number of experiments required^11^.

Although previous studies have reported the effects of different extraction solvents on the phytochemicals and bioactivity of plant extracts, none have developed extraction kinetic and phenomenological models for the effects of temperature variations and different common solvents (methanol, ethanol, and water) on the antioxidant and antimicrobial capacity, inhibitory effect on cancer cell proliferation, and extraction efficiency of polyphenolics from *O. gratissimum*.

## Materials and methods

### Chemicals and instruments

Mueller–Hinton agar (MHA), 1-diphenyl-2-picrylhydrazyl (DPPH), Mueller–Hinton broth (MHB), methanol, ethanol, sodium carbonate, trolox, and Folin–Ciocâlteu reagent were purchased from Merck & Co. (Kenilworth, NJ, USA). The instruments used include the gas chromatography-mass spectrometer (GCMS-QP2010 SE, Shimadzu Corporation, Kyoto, Japan), an ultraviolet–visible spectrophotometer (UV-2450; Shimadzu Corporation, Kyoto, Japan), Soxhlet apparatus, rotary evaporator, microscope (Leica DFC295), and cylinder crusher (XPC100; Tencan, Changsha County, China). *O. gratissimum* plant specimens were collected from the southern part of Nigeria. Identification of the plant was performed by the Department of Plant Science and Biotechnology, Abia State University, Uturu, Nigeria.

### Preparation of the extracts

Prior to extraction, the plant material was dried indoors in the dark and subsequently crushed to achieve an average particle size of 3–5 mm. The same particle size range was targeted for all extraction processes.

For the *O. gratissimum* ethanolic (OGE), methanolic (OGM), and water (OGW) extractions, 100 g of the crushed *O. gratissimum* leaves were placed in a Soxhlet apparatus. Following the addition of the solvent (600 mL), and the leaves were extracted for 6 h at 40 °C, 50 °C, and 60 °C for ethanolic and methanolic extractions. For extraction with water, the temperature used were 90 °C, 100 °C, and 110 °C. The extraction temperatures for each solvent were chosen based on the boiling temperature of the solvent. After extraction, a rotary evaporator was used to recover the crude extract, which was freeze-dried prior to storage in a refrigerator (4 °C) until use.

### Gas chromatography–mass spectrometry analysis of *O. gratissimum* extracts

The chemical composition of the extracts was determined using gas chromatography–mass spectrometry (GC–MS), in a method modified from that reported by Kavaz et al.^5^. A fused-silica capillary column (film thickness: 30 × 0.25 HP-5Ms, 0.25 µm) was used for the determination of compounds, with helium acting as the carrier gas (1/mL flow rate). An oven temperature of 40 °C was set and held for 5 min, then increased by 3 °C/min up to 270 °C. The split ratio was set at 60:1. The connection parts and ion sources were set at a temperature of 180 °C, and an interface temperature of 240 °C was set for the mass spectrometer. For the ionization energy amount, 70 eV was adopted, while the electron impact (EI) mode was chosen to produce stable and reproducible mass spectra and a value range of 50–650 m*/z* was used for the running of samples. The MS delay time before scanning was 5 min. The identification of the extract components was performed by comparing each compound’s mass spectra with records in the National Institute of Standards and Technology (Gaithersburg, MD, USA) 14 and Wiley (Hoboken, NJ, USA) MS libraries.

### Determination of total phenolic content

The total phenolic content (TPC) of the extracts was evaluated using the Folin–Ciocâlteu technique as described by Shahidi and Zhong^6^, with a few modifications. Briefly, 100 µL (0.5 mg/mL) of the OGE, OGM, and OGW extracts was placed in separate tubes. Next, 500 µL (1% v/v) of Folin–Ciocâlteu reagent was added to each tube and the tubes were gently agitated for 5 min. Subsequently, 400 µL (20% w/w) of sodium carbonate was added to the aliquots, which were then incubated in the dark at room temperature for 20 min. The absorbance of each mixture was evaluated with an ultraviolet–visible spectrophotometer at 765 nm. Sodium carbonate solution without any addition of Folin–Ciocâlteu reagent were used as blanks. For the calibration curve, gallic acid standards were used. TPCs of the samples were determined from the linear regression of the gallic acid standards. The results were represented as the gallic acid equivalent (GAE) per gram of dry weight of *O. gratissimum* extract (mg GAE/g). The procedure was conducted in triplicate (n = 3).

### DPPH free radical scavenging assay

The free radical scavenging activity of each extract was determined according to the methods described by Rakmai et al.^12^, with slight modifications. In brief, 2 mL of DPPH–methanol solution (180 µmol/L) was mixed with the different extracts (0.1 mg/mL). The aliquots were incubated in the dark at 25 °C. The absorbance of each sample was determined using a spectrophotometer at 517 nm at different time intervals (0–60 min). Aliquots of the extracts without addition of DPPH–methanol solution were used as blanks. Trolox, a synthetic analog of vitamin E, was used as a positive control. Equation (1) was used to evaluate the scavenging properties of the extracts. The procedure was carried out in triplicate (n = 3)

1 $$ {\text{\% }}\;{\text{DPPH}}_{{{\text{scavenging}}}} = \left[ {\frac{{\left( {{\text{A}}_{{{\text{extract}}}} - {\text{A}}_{{{\text{blank}}}} } \right){ }}}{{{\text{A}}_{{{\text{control}}}} )}} \times 100} \right] $$

where A_sample_ is the extract + DPPH, A_blank_ is the extract only, A_control_ is the absorbance of the control solution (containing only DPPH).

### Mathematical modeling of antioxidant activity

To generate a description of the process, some assumptions were made, including:

1. The particles are solid, with a uniform distribution of bioactive components within the sphere.
2. Absolute miscibility of solvent, with negligible liquid phase transfer resistance.
3. The migrations of bioactive components in plant samples are according to the coefficient of diffusion (Cd) and independent of time.
4. The bioactivity of the plant chemical constituent in the solvent is time-dependent.

To determine the antioxidant kinetic pattern of the *O. gratissimum* extracts, the general kinetic models of zero-, first-, and second-order reactions were adopted, as presented in Eqs. (2)–(4), respectively^13^

2 $$\left[{C}_{0}\right]-\left[C\right]= kt $$

3 $$\left[C\right]=\left[{C}_{0}\right]\mathrm{exp }(-kt)$$

4 $$\frac{1}{[C] }-\frac{1}{\left[{C}_{0}\right]}= kt$$

where *t* is the reaction time (day); *k* (/day) represents the reaction constant; [*C*_0_] and [*C*] are the initial and final amounts of compounds, respectively, at the different times *t* and temperatures (°C).

### Calculation of phenolic content coefficient and relative antioxidant activity index

The relative antioxidant capacity (RACI) was calculated to further compare the antioxidant activity of the different extracts according to methods by Gorjanovic et al.^14^. RACI was calculated by subtracting the antioxidant mean values of the extracts from the raw data divided by the standard deviation. The formula for calculating the RACI is presented in Eq. (5)

5 $$\mathrm{RACI }=\frac{\mathrm{y}-x }{\sigma }$$

where σ represents the standard deviation, y raw data, and *x* the mean. The phenolic antioxidant coefficient (PAC) were calculated as the ratio between the total phenolic content and the antioxidant capacity of the extracts^15^.

### Evaluation of antibacterial activity of O. gratissimum extracts

Two strains of Gram-negative bacteria, *S. typhimurium* (ATCC 13311) and *E. coli* (ATCC 8739), and two strains of Gram-positive bacteria, *B. cereus* (ATCC I4579) and *S. aureus* (ATCC 25923), were obtained in suspension from the American Type Culture Collection (Manassas, VA, USA). All were adjusted to 1.5 × 10^8^ CFU/mL which is the McFarland standard, and examined to discern their minimum inhibitory concentration (MIC) and minimum bactericidal concentration (MBC) relative to OGE, OGM, and OGW extracts using the microdilution method described by Devrnja et al.^16^, with some modifications. Briefly, extracts were diluted to concentrations of 500–2 mg/mL and added to the micro-well plates containing MHB. Next, 10 µL of the McFarland standardized microbial suspension inoculums were added to all of the wells for each type of bacteria strain, and the plates were then incubated for 24 h at 37 °C. The MIC of the samples were established following the addition of 40 µL of iodonitrotetrazolium chloride (INT; 0.02% m/v) and incubation for 30 min at 37 °C. The lowest concentration having no form of microbial growth (*P* ≤ 0.005) in comparison with the positive control (culture medium containing Tween 80, microbial suspension, and ethanol) was established as the MIC. Culture medium (MHB) containing only the different types of bacteria strain served as the negative control. To further establish the MIC, the bacteria in the culture medium showing no microbial growth were transferred to an MHA plate and then incubated at 37 °C for 24 h. The lowest concentrations of the *O. gratissimum* extracts (OGE, OGM, and OGW) that inhibited the proliferation of the test microorganism after 24 h of incubation at 37 °C were reported as the respective MBCs. All experimental procedures were conducted in triplicate (n = 3).

### Trypan blue exclusion assay

The determination of the cytotoxic properties of *O. gratissimum* extracts on MDA-MB-231 cells was carried out using a trypan blue dye exclusion assay. Cells (3 × 10^4^/mL) were plated in 35 mm dishes and allowed to incubate overnight before treatment with several concentrations of extract (0–100 µg/mL) according to the respective OGE-40, OGE-50, OGE-60, OGM-40, OGM-50, OGM-60, OGW-90, OGW-100, and OGW-110 protocols. The control group was treated with 1 mL of Dulbecco’s modified eagle medium. After a 48-h incubation period, trypan blue dye (4%) was dropped into all culture plates, which were further incubation for 40 min. The viability of the cells was determined using an inverted microscope (DFC295; Leica Camera, Wetzlar, Germany), choosing cells from 30 randomly selected sites within the dishes.

### Statistical analyses

All experiments were conducted a minimum of three times (n ≥ 3). One-way analysis of variance (ANOVA) and Student’s two-tailed *t* test were used for statistical comparisons. Differences in mean values were regarded as very significant at *P* ≤ 0.0001, significant at *P* ≤ 0.05, and nonsignificant at *P* > 0.05. The Statistical Package for the Social Sciences version 23 software program (IBM Corporation, Armonk, NY, USA) was used to perform statistical analysis and computations. The OriginPro version 2016 software program (OriginLab Corporation, Northampton, MA, USA) was used to create graphical representations of results.

## Results and discussion

### Gas chromatographic chemical composition analysis of O. gratissimum extracts

The extraction procedure is one of the most important steps in the use of natural resources, as it can affect the chemical makeup as well as the biological properties of extracts obtained. Several methods of extraction rely on different analyte mechanisms of isolation from matrices, and the technique used may rely on the nature of the plant material. For this study, the chemical profiles of three different extracts (OGE, OGM, and OGW) were revealed using GC–MS. The results obtained for the OGE are presented in Table 1, which shows that the lowest concentration (% wt/wt) of observed components was found in extracts prepared at 50 °C (OGE-50), while the concentrations were higher in extracts prepared at 40 °C (OGE-40) and at 60 °C (OGE-60). Samples obtained using ethanolic and methanolic extracts contained a higher amount of bioactive compounds, including sabinene, terpenene, thymol, copaene, caryophyllene, humulene, selinene, caryphylene oxides, and phytol, compared to OGW extracts. The concentration of constituent compounds in OGE extracts varies at different temperatures, with OGE-60 > OGE-40 > OGE-50. This might be due to altered dissolving abilities of the solvents at different temperatures during the process of extraction^17^. A study by Cvetanović et al.^18^ on extractions of *Aronia melanocarpa* M. stem reported more flavonoids and phenolic content in extractions with ethanol compared to extractions with methanol. In contrast, our results revealed that a high number of compounds was observed in OGM extracts in all cases (Table 2). Hence, it can be generalized that extraction with methanol is the preferred technique for isolation of several bioactive compounds from the *O. gratissimum* plant. As reported by Gharaati^19^, methanol can easily infiltrate into plant tissue and increase the process of extraction. The concentration of compounds in the methanolic extracts was highest in OGM-40, followed by OGM-60 and OGM-50. For OGW extracts, those prepared at 110 °C showed the highest number and concentration of identified compounds, as reported in Table 3. The differences in concentration of compounds could be a consequence of the extraction temperature resulting in rupture of the plant cell walls, leading to diffusion of the plant constituents into the water medium. Moreover, the lower number of bioactive compounds in OGW extracts compared to OGE and OGM extracts might be due to reduced compound solubility in water, as well as the extraction conditions. Nevertheless, an increase in temperature causes a decrease in water’s dielectric constant, and as a result, fewer polar compounds will be dissolved in it. Chemical and physical properties of water can change drastically under supercritical conditions. In addition, under certain operational conditions, the polarity of water can be fine-tuned^18^.

**Table 1:** Chemical composition of *O. gratissimum* ethanolic extract.

Retention time (min)	Chemical name	Content (%, wt/wt)		
		OGE-40	OGE-50	OGE-60
15.692	Sabinene	0.48	0.51	0.74
19.858	γ-Terpinene	4.61	3.21	5.02
33.017	Thymol	2.17	1.07	2.23
35.7	α-copaene	0.53	0.93	1.47
37.575	Trans-caryophyllene	1.51	1.12	1.96
39.2	α-Humulene	0.88	1.28	0.54
40.233	β-Selinene	4.41	3.65	4.89
44.65	Caryophyllene oxide	2.01	1.67	2.28
55.183	Trans-phytol	0.19	0.19	0.19

**Table 2:** Chemical profiling for methanolic extract of *O. gratissimum.*

Retention time (min)	Chemical name	Content (%, wt/wt)		
		OGM-40	OGM-50	OGM-60
5.092	Methyl ester	0.04	0.15	0.18
5.742	Glycerin	0.45	0.52	0.45
18.142	Cymene	4.66	4.68	4.65
32.275	Thymol	0.13	0.16	0.24
37.917	Trans-caryophyllene	0.39	1.34	0.42
40.975	β-Selinene	0.29	0.31	0.96
42.092	Phenol, 3-(1,1-dimethylethyl)-4-methoxy-	0.70	0.85	0.87
55.4	Phytol	0.51	1.21	0.43
56.033	Trans-phytol	0.96	0.92	1.12
60.425	Phenol, 2-methyl-5-(1-methylethyl)-(CAS) carvacrol	0.85	0.52	0.89

**Table 3:** Chemical composition of *O. gratissimum* water extract.

Retention time (min)	Chemical name	Content (%, wt/wt)		
		OGW-90	OGW-100	OGW-110
5.075	1,2-Butanediol	0.09	0.07	0.12
28.825	N-Acetyl-proline	4.40	2.92	4.85
32.425	Phenol, 5-methyl-2-(1-methylethyl)	2.53	1.41	3.06
37.867	Caryophyllene	0.29	0.13	0.41
40.892	β-Selinene	0.72	0.64	0.94
44.975	Caryophyllene oxide	4.31	3.31	4.86
54.367	Neophytadiene	4.39	4.39	4.39
55.9	2-Hexadecen-1-ol, 3,7,11,15-tetramethyl-,[R-[R*,R*-(E)]]	4.38	1.38	4.26

### Total phenolic content of O. gratissimum extracts

TPC yield is essential, especially for industrial applications at large scale and for economic purposes. Thus, this study aimed to identify the extraction technique generating the highest TPC yield. Phenolic profiling of extracts was established by applying the Folin–Ciocâlteu method (Table 4). Significant differences (*P* < 0.05) in TPC were observed for the extractions obtained using the different methods. The amount of TPC obtained in OGM-40 (2.43 ± 0.24 mg GAE/100 g) was approximately 50% higher than the corresponding value obtained from the rest of the extracts, while this value was much lower in the case of OGW-100. At a temperature of 110 °C, however, OGW exhibited much higher (*P* < 0.05) TPC yields than OGE-40, OGE-50, and OGM-50. Similar findings were reported by Fan et al.^20^. OGM-40 showed the highest yield of phenolic content and is hence considered to be the most effective phenolic extraction technique for *O. gratissimum*. This could be due to the high polarity and subcritical dissociation of bonds of methanol compared to other types of solvent during phenolics recovery from this plant. With regards to the different extraction techniques, our findings are in line with those of the study by Veličković et al.^21^. The *O. gratissimum* phenolic content has been well established in the literature; however, several factors, such as harvest season, type of fertilization, habitat/location, and plant malnutrition, can affect the phenolic content in *O. gratissimum*^22^. Literature data suggest that *O. gratissimum* contains high levels of polyphenols, flavonoids, and fatty acids^23,24^. To our knowledge, this study is the first to report a comprehensive analysis of the effect of temperature and solvent type on the TPC of *O. gratissimum*.

**Table 4:** Content of phenolics from different *O. gratissimum* extracts.

Extracts	TPC (mg GAE/100 g)
OGE-40	1.38 ± 0.26^a^
OGE-50	1.36 ± 0.32^a^
OGE-60	2.29 ± 0.31^b^
OGM-40	2.43 ± 0.24^c^
OGM-50	1.41 ± 0.21^a^
OGM-60	1.82 ± 0.74^c^
OGW-90	1.26 ± 0.44^a^
OGW-100	1.15 ± 0.52^d^
OGW-110	1.80 ± 0.61^c^

Values shown are mean of three replicates (n = 3) ± SD. Values with different superscript lowercase letter (a–d) within each column are significantly different (*P* ≤ 0.05).

### Antioxidant activities of O. gratissimum extracts

**Figure 1:**
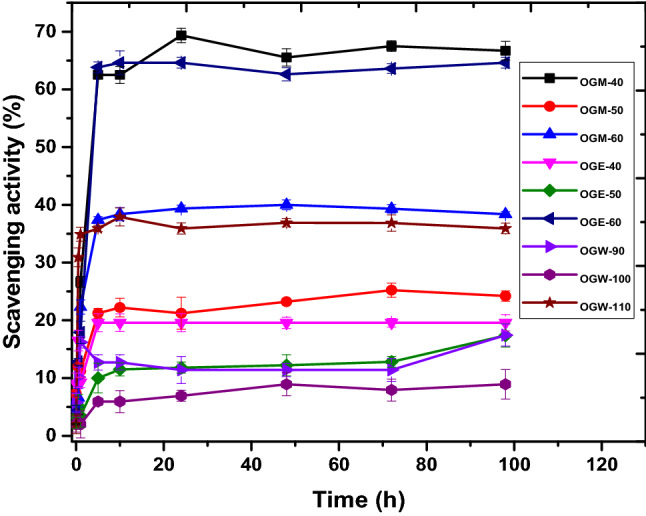
Antioxidant activity of *O. gratissimum* extract (results are mean values of three replicates [n = 3] expressed in percentage; bars represent standard deviations).

In a biological system, free radicals can cause damage in vivo and are consequently considered a cause of numerous diseases. Hence, the scavenging of free radicals is an essential task for antioxidant compounds to protect living systems. The results of our experiments testing antioxidant activity revealed that the extracts investigated showed notable potential for oxidation inhibition. As shown in Fig. 1, the activity can be ranked as follows: OGM-40 > OGE-60 > OGW-110 > OGM-50 > OGE-40 > OGE-50 > OGW-90 > OGW-100. The free radical-scavenging activity of compounds, as outlined by Kfoury et al.^24^, is examined after 30 min contact of DPPH solution with the compound. However, Kamimura et al.^25^ proposed that the antioxidant activity of compounds is likely to last much longer, depending on the oxidation kinetics over time. OGM antioxidant ability was higher from the period of 1–5 h compared to the rest of the extracts. The high antioxidant activity of OGM-40 is attributed to the high level of phenolics in this extract compared to the others. The findings of this study are in agreement with those of the study by Ngo et al.^26^ on antioxidant activity of methanolic extracts of *Salacia chinensis* L. root.

Among the OGW extracts, the scavenging power was the greatest at 100 °C, less at 90 °C, and even more reduced at 110 °C. This shows that antioxidant properties can be greatly reduced at extraction temperatures above the optimum/boiling temperature of the solvent, as this can lead to the denaturation of phenolics, as was the case with OGW-110.

For the OGE and OGM extracts, the effect of temperature on the oxidant-scavenging properties of these extracts was in line with the findings of Molaveisi, Beigbabaei, Akbari, Noghabi, and Mohamadi^27^.

The DPPH scavenging capacity of the extracts was also evaluated by calculating their IC_50_ values, which correlates to the amount of extract that is capable of scavenging 50% of the free radicals contained in the reaction mixture. A low IC_50_ value indicates high free radical-scavenging activity, and vice versa. The IC_50_ values of the extracts are within the range of 0.75–2.84 mg/mL (Table 5). Based on the IC_50_ values, the order of free radical-scavenging activity of the extracts is as follows: OGE-40 > OGE-50 > OGE-60 > OGW-90 > OGM-50 > OGM-40 > OGM-60 > OGW-100 > OGW-110 (Table 5). The high antioxidant capacity of *O. gratissimum* is often considered a function of its high phenolic content. However, our results showed that extracts with low TPC also exhibited high antioxidant activity, suggesting that in addition to phenolics, other factors, such as extraction temperature and solvent type, can also contribute to an extract’s antioxidant activity.

**Table 5:** Half-maximal concentration (IC_50_) values for the DPPH free radical-scavenging activity assay of *O. gratissimum* extracts.

Extracts	Scavenging activity (IC_50_; mg/mL)
OGE-40	0.55 ± 0.10^a^
OGE-50	0.72 ± 0.11^b^
OGE-60	0.83 ± 0.01^c^
OGM-40	1.76 ± 0.01^b^
OGM-50	1.14 ± 0.41^c^
OGM-60	1.84 ± 0.02^b^
OGW-90	0.81 ± 0.08^b^
OGW-100	1.86 ± 0.03^b^
OGW-110	1.95 ± 0.09^b^

Values shown are means of three replicates (n = 3) ± SD. Values with different superscript letters (a–c) within each column are significantly different (*P* ≤ 0.05).

### Phenolic antioxidant coefficient and relative capacity index

**Figure 2:**
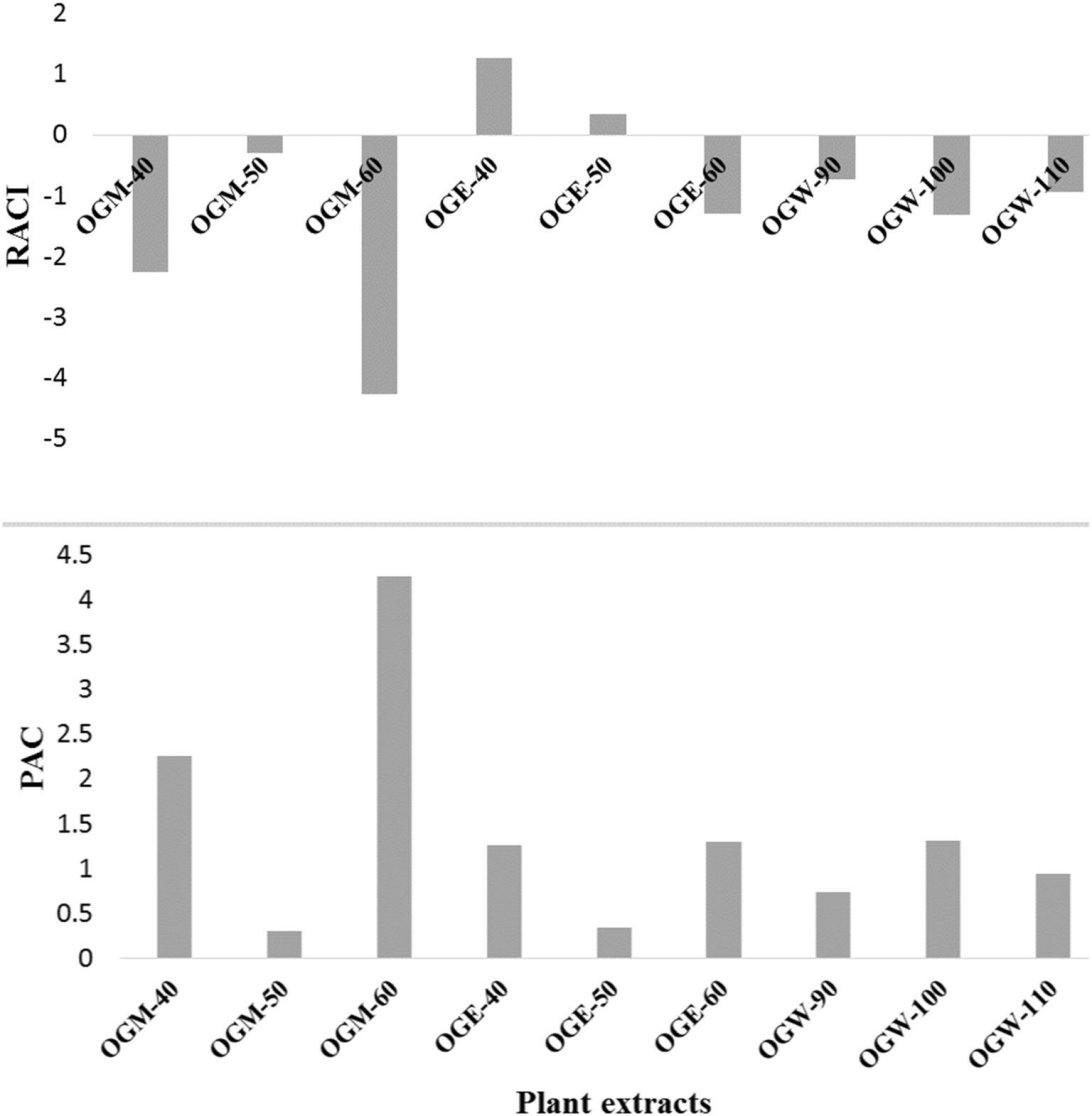
Phenolic antioxidant coefficient (PAC) and relative antioxidant capacity index (RACI) of *O. gratissimum* extracts.

In order to further compare the TPC and antioxidant capacity of the extracts, two additional parameters, namely, PAC, which is the ratio between TPC, and particular antioxidant capacity; and RACI, which determines the total reducing capacity, were introduced. In Fig. 2, it can be seen that the highest RACI value is ascribed to OGE-40 (1.26), followed by OGE-50 (0.34). Negative RACI values were seen for OGM-40 (− 2.25), OGM-50 (− 0.30), OGM-60 (− 4.26), OGE-60 (− 1.30), OGW-90 (− 0.73), OGW-100 (− 1.31), and OGW-110 (− 0.94). Though RACI and TPC show the antioxidant capacity of phenolics to a certain extent, PAC compares the efficiencies of phenolic in the samples. Interestingly, the lowest PAC values are seen for extracts with positive RACI values (i.e., high antioxidant capacity), while extracts with the most-negative RACI values possess higher PAC values (Fig. 2). A study by Wojdylo et al.^28^ suggest that with high PAC values are not a reflection of high antioxidant activity in plants; however, plants with substantial PAC and RACI values is an indication of their rich antioxidant potential. The findings of this study are similar to those from a study by Petrovic et al.^29^.

### Kinetic models for the study of the effect of solvent composition and temperature on the antioxidant activity of *O. gratissimum* extracts

**Figure 3:**
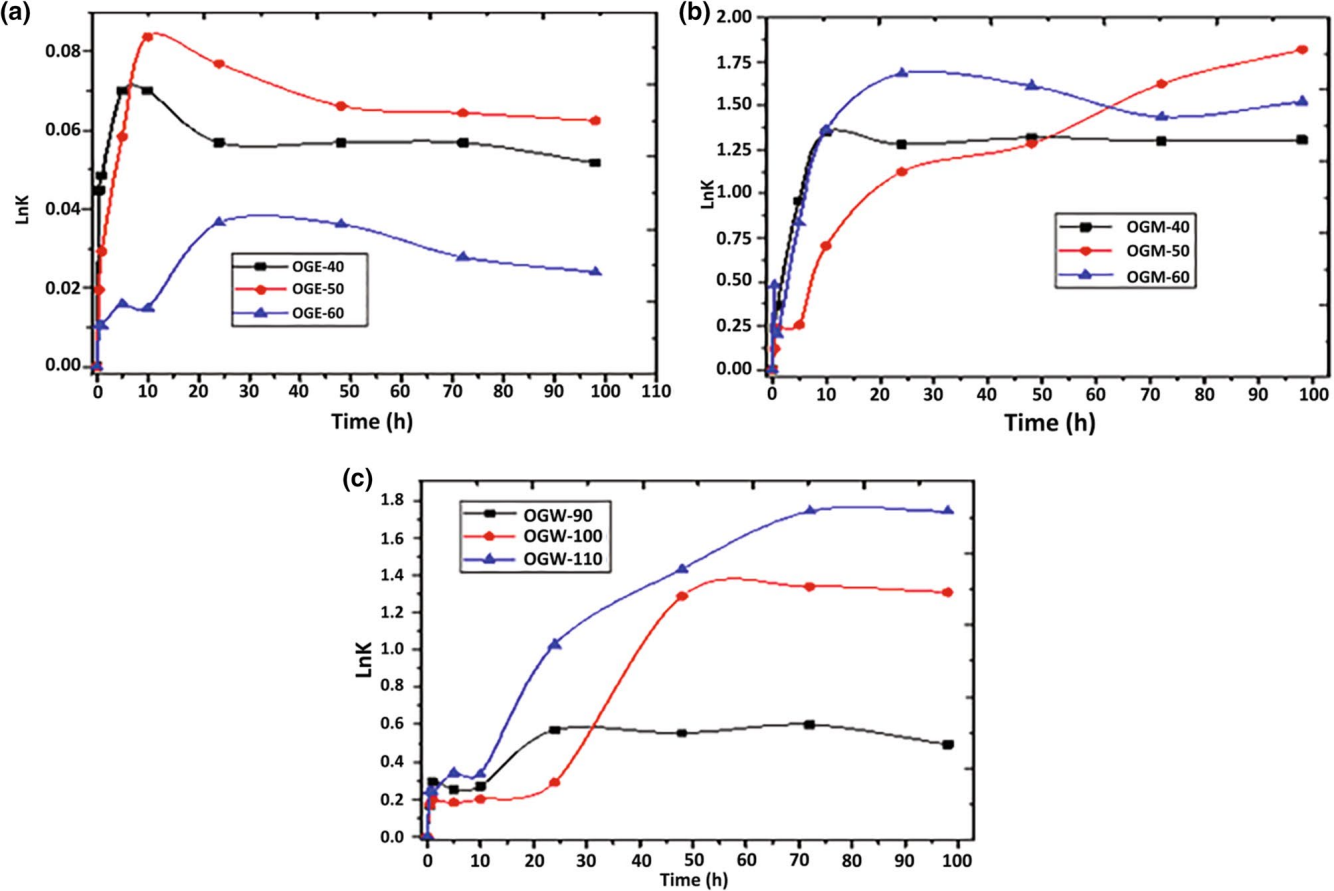
Arrhenius plots for the DPPH radical-scavenging activity of *O. gratissimum* extracts at different extraction temperatures as a function of exposure time. (**a**) Ethanolic extracts, (**b**) methanolic extracts, (**c**) water extracts. Bars represent mean ± standard deviation.

As shown in Fig. 3, varying the temperature and type of solvent had a significant effect on the antioxidant kinetics of *O. gratissimum* extracts. Data analysis using several kinetic models showed that the highest coefficient of determination (r^2^) value for both the zero- and first-order kinetic models was achieved with OGE-50, with r^2^ values of 0.694 and 0.793, respectively. In contrast, for the second-order model, the highest adjusted r^2^ value was achieved with OGE-60 (r^2^ = 0.436, K = 0.314; Table 6). The linear relationship between lnK for the antioxidant activity for all the extracts over time had the best fit to the second-order kinetics model. These results are in agreement with previous studies, such as a study that reported the kinetics of the effect of temperature on antioxidant activity of Iranian jujube honey^27^. The kinetic parameter rate constant (K) for the antioxidant activity of *O. gratissimum* showed a strong temperature dependence for the second-order model, as K tends to increase with an increase in temperature. For example, OGW at the temperatures of 90 °C, 100 °C, and 110 °C had K values of 0.081, 0.084, and 0.246, respectively. This result indicates that extractions at higher temperatures lead to a faster decrease in antioxidant activity. Similar results were observed for ascorbic acid in ground cashew apple^30^ and anthocyanins in blackberry juice and its concentrate^31^.

**Table 6:** Kinetic parameters of DPPH radical-scavenging activity of *O. gratissimum* extracted with different solvents at different temperatures as a function of exposure time.

Sample	Zero order		First order		Second order	
	r^2^	K_0_	r^2^	K_1_	r^2^	K_2_
OGM-40	0.378	0.005	0.032	0.001	0.242	0.019
OGM-50	0.270	0.001	0.431	0.001	0.2387	0.034
OGM-60	0.494	0.001	0.453	0.001	0.202	0.084
OGE-40	0.192	0.001	0.189	0.001	0.148	0.037
OGE-50	0.694	0.001	0.793	0.001	0.409	0.17
OGE-60	0.657	0.001	0.083	0.001	0.436	0.314
OGW-90	0.325	0.002	0.090	0.06	0.237	0.081
OGW-100	0.339	0.004	0.433	0.001	0.202	0.084
OGW-110	0.259	0.002	0.320	0.001	0.203	0.246

*r*^*2*^ regression coefficient, *K*_*0*_ rate constant for zero-order reaction, *K*_*1*_ rate constant for first-order reaction, *K*_*2*_ rate constant for second-order reaction.

### Antibacterial activity of *O. gratissimum* extracts

The MIC and MBC measured for each extract are presented in Table 7. For each extract, the MIC was within the 5–150 μg/mL range, which, according to a study by Kavaz et al.^5^, indicates very good antibacterial activity. The highest sensitivity to *O. gratissimum* extract was demonstrated by *S. aureus*, while *S. typhimurium* was the most resistant. A significant difference (*P* < 0.05) in antimicrobial activity was observed for all of the extracts when compared to the negative control. The OGM-40 extract had a significantly higher antibacterial activity for all bacteria tested compared to the rest of the extracts. For *S. aureus*, particularly high antibacterial activity was observed, as the MIC value was 5 μg/mL. The rest of the bacteria strains also showed high sensitivity to OGM extracts. For OGE-40, the lowest MBC was against *B. cereus* (150 μg/mL). OGE-50, OGW-90, and OGW-100 had the same MBC values for all bacteria (250 μg/mL, 250 μg/mL, and 300 μg/mL, respectively), while the highest antibacterial activity for OGM-60 was against *E. coli* and *B. cereus* (100 μg/mL). In addition, for OGE-60, the highest antibacterial activity was observed for *S. aureus* (40 μg/mL), and for OGM-50 and OGW-100, the highest antibacterial activity was against *B. cereus*, at 100 μg/mL and 80 μg/mL, respectively. Although the OGW samples showed the lowest antibacterial properties, they still showed antimicrobial activity at higher concentrations. In general, antimicrobial activity of the different extracts of *O. gratissimum* may be related to the phytochemical composition^5^, extraction techniques, and multiple mechanisms of action^32^.

**Table 7:** Minimum inhibitory and bactericidal concentration (MIC, MBC) against *E. coli*, *B. cereus*, *S. aureus*, and *S. typhimurium* for *O. gratissimum* extracts.

Antibacterial compound	*E. coli*		*B. cereus*		*S. aureus*		*S. typhimurium*	
	MIC (µg/mL)	MBC (µg/mL)	MIC (µg/mL)	MBC (µg/mL)	MIC (µg/mL)	MBC (µg/mL)	MIC (µg/mL)	MBC (µg/mL)
OGE-40	100 ± 5.06^aA^	200 ± 9.69^aB^	100 ± 10.4^aA^	150 ± 7.54^aC^	150 ± 5.31^aC^	200 ± 7.84^aB^	250 ± 4.32^aD^	300 ± 8.36^aE^
OGE-50	140 ± 8.43^bA^	250 ± 7.83^bB^	140 ± 6.84^bA^	250 ± 5.62^bB^	140 ± 7.64^aA^	250 ± 10.32^bB^	140 ± 9.34^bA^	250 ± 9.71^bB^
OGE-60	20 ± 2.89^cD^	60 ± 5.5^cB^	40 ± 5.67^cC^	60 ± 6.45^cB^	20 ± 2.73^bD^	40 ± 4.67^cC^	60 ± 5.82^cB^	100 ± 9.54^cA^
OGM-40	20 ± 2.65^cA^	40 ± 9.21^ dB^	10 ± 1.65^dC^	20 ± 2.51^dA^	5 ± 0.56^cD^	20 ± 3.21^dA^	40 ± 4.84^ dB^	80 ± 9.64^dE^
OGM-50	100 ± 6.52^aE^	150 ± 5.67^eC^	80 ± 5.4^eD^	100 ± 6.46^eE^	80 ± 6.94^dD^	140 ± 7.69^eC^	200 ± 4.75^eB^	300 ± 8.57^aA^
OGM-60	60 ± 3.56^dA^	100 ± 6.73^fB^	60 ± 4.56^fA^	100 ± 4.56^eB^	100 ± 7.65^eB^	150 ± 9.73^fC^	100 ± 8.53^fB^	200 ± 9.78^eF^
OGW-90	140 ± 4.96^bC^	250 ± 5.78^bA^	140 ± 5.65^bB^	250 ± 6.83^bA^	140 ± 7.58^aC^	250 ± 8.52^bA^	140 ± 4.78^bB^	250 ± 9.45^bA^
OGW-100	150 ± 8.56^bA^	300 ± 7.63^gB^	150 ± 4.65^bA^	300 ± 6.43^fB^	150 ± 6.14^aA^	300 ± 9.12^gB^	150 ± 6.14^bA^	300 ± 9.14^aB^
OGW-110	80 ± 5.67^eC^	150 ± 7.95^eE^	40 ± 4.41^cD^	80 ± 5.65^gC^	100 ± 5.27^fC^	250 ± 8.23^bB^	150 ± 6.43^aB^	300 ± 9.12^aA^

Values shown are means of three replicates (n = 3). Values with different superscript lowercase letter (a–g) for MIC and MBC data within each column are significantly different (*P* ≤ 0.05). Values with different superscript uppercase letter (A–E) within each row are significantly different (*P* ≤ 0.05).

### Cytotoxic activity assay

**Figure 4:**
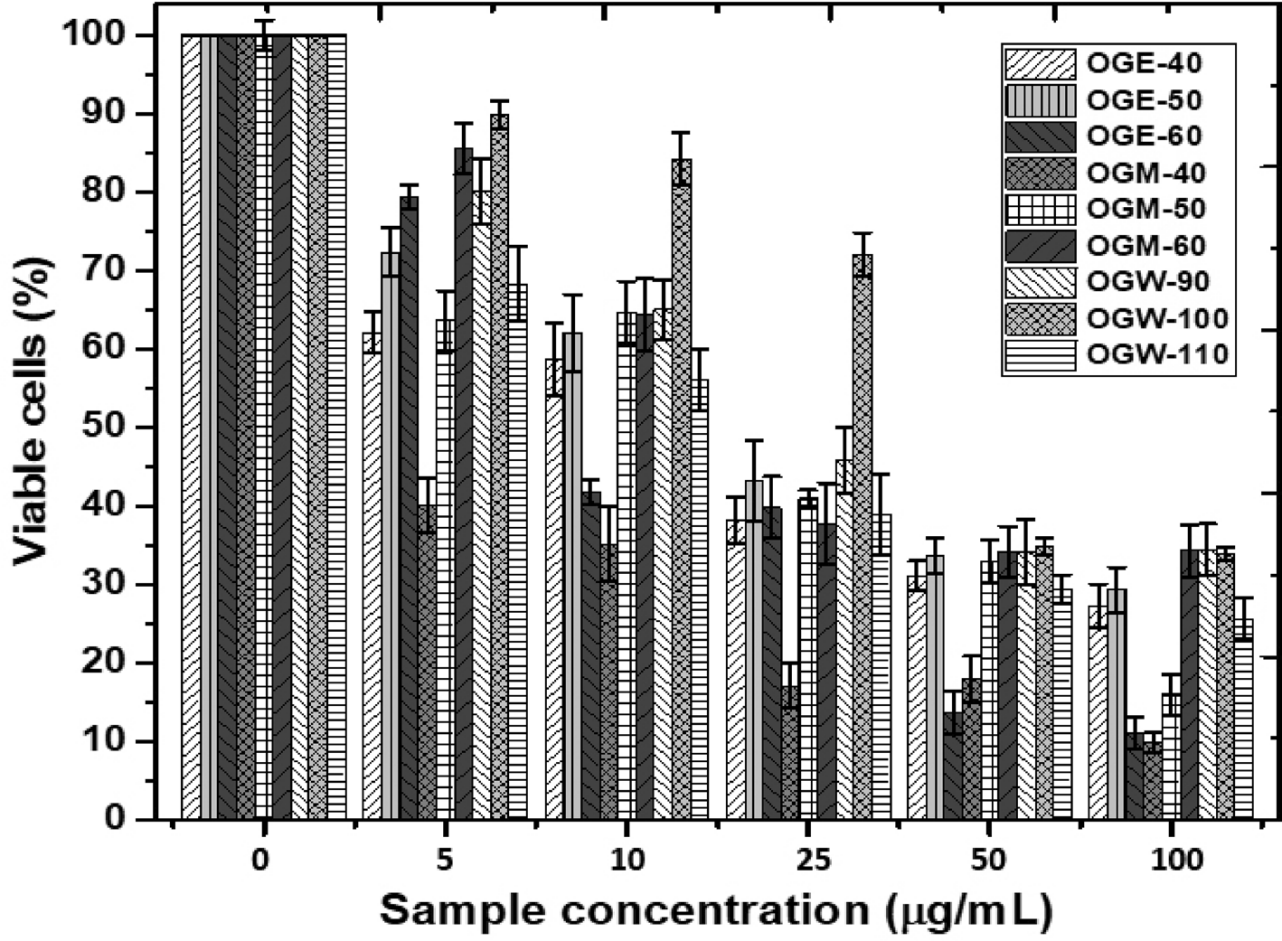
Effect of 48 h treatment with *O. gratissimum* extracts on MD-MD-231 breast cancer cells. Bars represent mean ± standard deviation.

Exclusion assay with trypan blue dye revealed high toxicity of *O. gratissimum* on MDA-MB-231, a highly metastatic breast cancer cell line, after treatment with the extracts for a period of 48 h at various concentrations (0–100 µg/mL; Fig. 4). The results showed that cell viability declined with increases in extract concentration. The results of the experiments showed that OGM-40 inhibited the cell viability at a lower concentration compared to the rest of the extracts (*P* < 0.05, n ≥ 3; Fig. 4). The highest concentration (100 µg/mL) of OGE-40, OGE-50, OGE-60, OGM-40, OGM-50, OGM-60, OGW-90, OGW-100, and OGW-11 lowered the viability of the cell line to 27.22%, 29.32%, 11%, 9.83%, 15.97%, 34.25%, 34.47%, 33.86%, and 25.66% of the untreated control, respectively. The light microscope phase-contrast images for the trypan blue dye retention of the control group (untreated) and drug-treated cancer cells are shown in the supplementary data (Fig. S1). For the cancer cells treated with OGM-40, membrane blebs were also observed after 48 h of incubation. This is an indication of cell apoptosis, as suggested by Onyebuchi and Kavaz^7^. The high antiproliferative activity of OGM-40 compared to the rest of the extracts is likely a result of oxidative stress generation within the cell membrane.

## Conclusion

The chemical profile and bioactive properties of *O. gratissimum* extracts prepared using different extraction temperatures and solvent types were examined in this study. The results obtained herein suggest that both extraction temperature and solvent type have a significant effect on the phenolic content yield, antioxidant capacity, antimicrobial activity, and cancer cell cytotoxic properties of *O. gratissimum* extracts. For extracts prepared with methanol, extraction performed at 40 °C (OGM-40) was the optimal protocol for obtaining high TPC and desirable chemical properties. The extracts showed strong temperature dependence and best fit with the second-order kinetic model, as the rate of antioxidant activity tends to increase with an increase in extraction temperature. The findings of this study further establish the potential use of the extracts as alternative natural food antioxidants over synthetic preservatives. The antimicrobial and anticancer properties of the extracts also demonstrate their potential applications in the pharmaceutical and food industries.

## Supplementary Information

Supplementary Figure S1.
