# Supplementary material for: Effect of extraction temperature and solvent type on the bioactive potential of Ocimum gratissimum L. extracts
Source: Sci Rep. 2020 Dec 10;10:21760. doi: 10.1038/s41598-020-78847-5 (PMC7729875; doi:10.1038/s41598-020-78847-5)
Supplement: Supplementary file 1 — Supplementary Figure S1. [file 41598_2020_78847_MOESM1_ESM.docx]

**Effect of extraction temperature and solvent type on the bioactive potential of *Ocimum gratissimum* L. extracts**

**Confidence Onyebuchi ^a*^ Doğa Kavaz ^a,b^**

**^a^ Bioengineering Department, Faculty of Engineering, Cyprus International University, 98258, Northern Cyprus via Mersin 10 Turkey.**

**^b^ Biotechnology Research Centre, Cyprus International University, Nicosia 99258, Northern Cyprus via Mersin 10 Turkey.**

***Corresponding author:** [**oukaeje@ciu.edu.tr**](mailto:oukaeje@ciu.edu.tr)

**
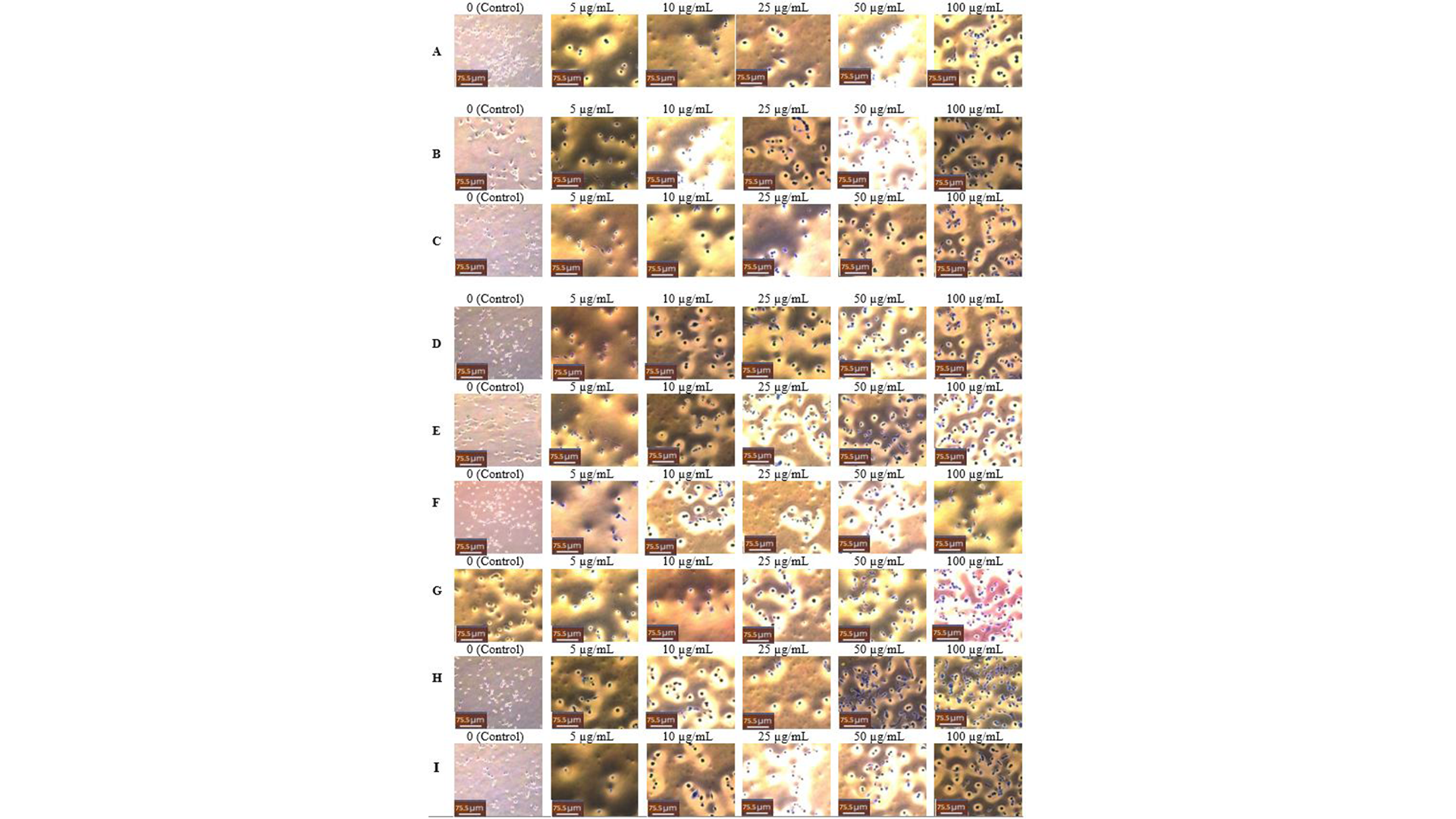
**

**Figure S1.** MDA-MB-23 cells Light microscope images (20x) of treatment with varying concentrations of samples: (**A**) OGE-40, (**B**) OGE-50 (**C**) OGE-60, (**D**) OGM-40, (**E**) OGM-50 (**F**) OGM-60, **(G**) OGW-90, (**H**) OGW-100, (**I**) OGW-110.

**Acronyms:** OGE, *Ocimum gratissimum* ethanolic extract; OGM *Ocimum gratissimum* methanolic extract; OGW, *Ocimum gratissimum* water extract. Scale bars = 75.5 μm.
